# Percutaneous debulking of tricuspid valve infective endocarditis vegetations using a large bore manual aspiration device — AlphaVac

**DOI:** 10.1016/j.jccase.2023.08.008

**Published:** 2023-08-26

**Authors:** Zach Rozenbaum, Ali Gholam, Frederick Helmcke, Asif Anwar, Anand Irimpen, Ali A. Alsaad

**Affiliations:** Department of Cardiology, Tulane University, New Orleans, LA, USA

**Keywords:** Percutaneous, Tricuspid, Infective endocarditis, Vegetations, AlphaVac

## Abstract

Most infectious endocarditis patients can be managed medically. However, non-responders to antibiotics and ongoing sequelae such as septic emboli, may require mechanical interventions. AngioVac (Angiodynamics, Latham, NY, USA) is a percutaneous aspiration device used for removal of thrombi, emboli, masses, and vegetations. Main drawbacks are the requirement for a perfusionist, two large-bore accesses, and meticulous de-airing. These drawbacks make the procedure more time-consuming and possibly increase the risk of complications. AlphaVac (Angiodynamics) omits the motor element, thereby overcoming several of the limitations. In the current report, we describe two cases of percutaneous aspiration of tricuspid valve vegetations using AlphaVac.

**Learning objective:**

To consider manual percutaneous aspiration of infective valvular vegetations using the AlphaVac cannula in case of insufficient response to antibiotics or for prevention of emboli.

## Introduction

**Fig. 1:**
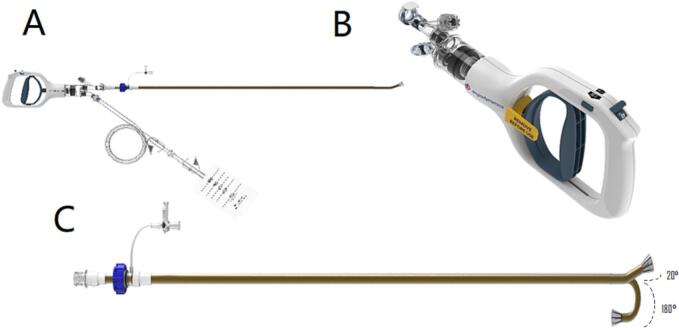
Components of the AlphaVac device (reproduced with permission from Angiodynamics) — the AlphaVac system is composed of a handle, waste bag, Y-connector with a 1-way valve, and the inner and outer cannula. (A) The assembled system; (B) the AlphaVac system handle — manual aspiration into the waste-bag is achieved by pressing the lever. A volume limiting switch can be toggled between 10- and 30 cm^3^ for each pull; (C) The 2 available cannulas differ by their maximal curvature — up to 20 and 180°. The inner cannula is preshaped to its maximal bend. Bending is achieved by exposing the inner cannula and its funnel-shaped tip.

Infectious endocarditis (IE) remains a major cause of mortality and imposes challenges in treatment. The leading cause of right sided IE is intravenous drug use (IVDU) [1]. While most patients can be managed medically, non-responders to antibiotics and ongoing sequelae such as septic emboli, may require mechanical interventions. The development of percutaneous mechanical aspiration devices for the purpose of vegetation debulking has introduced a new era of IE management. AngioVac (Angiodynamics, Latham, NY, USA) is a large bore percutaneous aspiration system indicated primarily for the removal of thrombi and emboli. However, it has been utilized to aspirate masses of uncertain nature as well as IE vegetations [2]. The device is composed of an aspiration cannula connected to centrifugal pump and console through tubing that maintains constant flow in a venous-to-venous system. Main drawbacks of the AngioVac system are the requirement for a perfusionist, the need for two large-bore accesses, the need for meticulous de-airing, and continuous maintenance of the de-aired system. These drawbacks make the procedure more time-consuming and possibly increase the risk of complications. AlphaVac (Angiodynamics) is a newer design for the aspiration system, with a similar cannula as AngioVac, but the aspiration mechanism is manual and achieved by pulling on the handle (Fig. 1). AlphaVac omits the motor element of the system, thereby overcoming several of the aforementioned limitations of AngioVac and simplifying the procedure. In the current report, we describe two cases of percutaneous aspiration of IE vegetations on the tricuspid valve (TV) using AlphaVac.

## Case report 1

A 31-year-old male with a history of IVDU, presented with dyspnea and chills. The patient was septic, developed renal failure (requiring dialysis) and anemia. Blood cultures showed methicillin resistant *Staphylococcus aureus* and he was started on antibiotics according to bacterial sensitivity testing. Chest computed tomography showed cavitary lesions, parenchymal opacities, and enlarged mediastinal lymph nodes. Echocardiography showed a large mobile mass on the tricuspid leaflet (the vegetation seemed to be adhered to posterior and/or septal leaflet), severe tricuspid regurgitation (TR), and bilateral severe ventricular systolic dysfunction. Transesophageal echocardiography (TEE) demonstrated a large mobile vegetation measuring 3.0 cm in diameter encompassing the septal and anterior leaflets as well as thickening of the chordae. The patient was at high risk for surgery due to multiorgan failure, including cardiac failure requiring vasopressors and inotropic support. Due to the persistent bacteremia for 18 days, percutaneous aspiration of the IE vegetation was planned for the patient.

On the operative TEE, the vegetation was better visualized on the 4-chamber and short-axis views than the bicaval view and appeared to be on the superior aspect of the valvular apparatus. Therefore, the vegetation appeared to be reachable from the femoral approach using the AlphaVac 180-degree cannula.

**Fig. 2:**
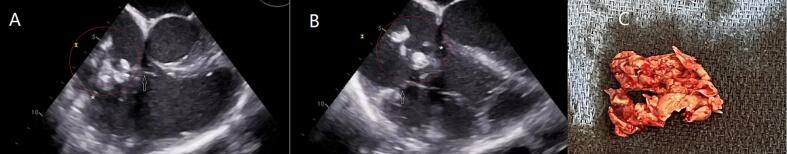
The vegetation on (A) and (B). Two-dimensional transesophageal echocardiography (scanning angles 85, and 0, respectively). Asterisk marks the location of the posterior leaflet and arrow marks the location of the anterior leaflet. (C) Vegetation's gross appearance after removal.

Bilateral femoral venous accesses were established, and a J-wire was advanced to the superior vena cava through a 6 Fr sheath under fluoroscopic guidance. Through an MPA catheter, the J-wire was exchanged for an extra-stiff Amplatz 0.035″ guidewire (Cook Medical, Bloomington, IN, USA), over which a 26 Fr DrySeal sheath (W.L. Gore & Associates Inc., Flagstaff, AZ, USA) was inserted after serial dilations and positioned in the inferior vena cava-right atrial junction and heparin was given for an activated clotting time target of above 300. In addition, access was obtained in the left femoral vein for blood return. The AlphaVac cannula was advanced to the tip of the sheath and prepared by connecting the handle and waste bag, and de-airing by allowing blood to backflow. Once in the right atrium, the funnel was expanded. Under TEE and fluoroscopic guidance, the cannula was navigated and positioned near the vegetation. Several manual suction attempts, initially 10 cm^3^ followed by 30 cm^3^, retrieved the entire visible vegetation without worsening the TR. The waste bag was periodically emptied during the procedure through an AngioVac filter, and the filtered blood was returned through the contralateral femoral sheath. A cutaneous suture was used for hemostasis. Fig. 2 shows the vegetation on TEE and its gross appearance after removal. Pre-procedure hemoglobin was 8.6 g/dl and 7.8 g/dl post-procedure. The blood cultures were cleared from bacterial growth after 3 days and the patient was extubated.

## Case report 2

A 47-year-old male with a history of intravenous heroin use and TV repair with annuloplasty ring due to IE 2 years previously. He presented to the emergency department with shortness of breath and lower extremity pain and was found to have septic shock due to methicillin sensitive *S. aureus*. Physical examination revealed signs of heart failure and gangrene of the toes. The patient had persistent bacteremia despite broad spectrum antibiotic therapy and lung parenchymal infarcts on computed tomography. Lower extremity doppler-ultrasound showed patent arteries and veins, and gangrene was presumed secondary to septic emboli to toes. TEE demonstrated moderate-severe TR, TV vegetation measuring 4.0 × 11.0 mm, and a patent foramen ovale. In a multidisciplinary discussion between internal medicine, cardiology, and cardiothoracic surgery specialists, and despite the relatively small vegetation, an attempt for percutaneous aspiration of the tricuspid vegetation was decided by consensus, mainly to prevent further embolization.

**Fig. 3:**
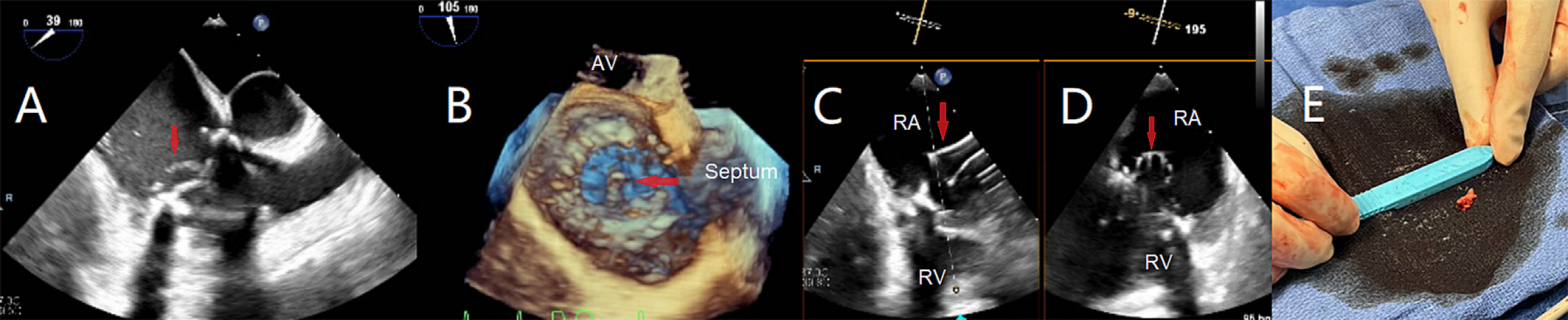
The vegetation marked by arrow on (A) two-dimensional transesophageal echocardiography (TEE); and (B) atrial perspective in three-dimensional TEE (AV, aortic valve); cannula in the right atrium viewed on X-plane after the aspiration was complete — (C) longitudinal; and (D) cross-sectional of the funnel (RA, right atrium; RV, right ventricle); (E) vegetation's gross appearance after removal.

Assessment by TEE showed a favorable angle by access through the superior vena cava and using the AlphaVac 180-degree cannula. Right internal jugular access and right femoral venous accesses were obtained. The remainder of the procedure was performed using similar techniques as specified above. The entire visible vegetation was retrieved without worsening of the TR. Fig. 3 shows the vegetation on 2D and 3D TEE, the cannula after the aspiration was complete, and the vegetation's gross appearance after removal. Pre-procedure hemoglobin was 9.3 g/dl and 8.7 g/dl post-procedure. The patient subsequently had clear serial blood cultures. Unfortunately, the gangrenes did not improve and eventually the patient underwent amputation.

## Discussion

This report describes for the first time the use of AlphaVac — a mechanical aspiration device in two cases of successful IE vegetation removal. The procedure can be performed in the catheterization laboratory and not necessarily in a hybrid operating room, and from our experience with the reported cases, the procedure is less time-consuming, efficacious, and possibly safer than AngioVac device.

Up to 12 % complications were reported using AngioVac, most of which were bleeding-related [3]. Another study describing 33 patients who underwent percutaneous aspiration for TV IE reported that 43 % had worsening TR [4]. Controlled suction may reduce the likelihood of suctioning unwanted structures such as leaflets or cords, thus better preserving valve integrity. Moreover, in instances of suctioning against a surface, such as vessel wall or right atrium, vacuum forms and with continuous aspiration more force is needed to retract the cannula. With AlphaVac the maximal aspiration can be reduced from 30 cm^3^ to 10 cm^3^, further limiting suction. As noted, in the current case reports TR did not worsen and there were no procedural complications. A helpful feature of the cannula to prevent injury is that the tip is readily detectible by its funnel shape, as opposed to other catheters which do not taper or expand, in which case it may be difficult to differentiate between the shaft and the tip of the catheter.

The benefit of a closed circuit is automatic return of filtered blood. There was a decrease in hemoglobin concentration in both cases, but anemia remained stable thereafter. The decrease in hemoglobin concentration is likely the result of hemodilution as well as mild procedural blood loss, although most of the aspirated blood was filtered and returned to the patient.

Periprocedural embolic complications were previously reported in 1–5.9 % of patients undergoing percutaneous aspiration using AngioVac [3,5]. In the current report there were no obvious peri-procedural embolic complications. Nevertheless, benefit in prevention of embolic complications is not expected when using AlphaVac compared to AngioVac.

## Conclusions

Our experience with the AlphaVac cannula, a manual and controlled percutaneous aspiration device, proved to be safe and efficacious for TV vegetation debulking. Further registry data might be beneficial to illustrate the benefits of this novel technique for TV vegetations percutaneous aspiration.

## Informed consent

Written informed consent was obtained from the patients.

## Declaration of competing interest

The authors declare that there is no conflict of interest.
